# Comparative Analysis of Deoxynivalenol Biosynthesis Related Gene Expression among Different Chemotypes of *Fusarium graminearum* in Spring Wheat

**DOI:** 10.3389/fmicb.2016.01229

**Published:** 2016-08-08

**Authors:** Chami C. Amarasinghe, W. G. Dilantha Fernando

**Affiliations:** Department of Plant Science, University of Manitoba, WinnipegMB, Canada

**Keywords:** *Fusarium graminearum*, wheat, trichothecenes, chemotype, qRT-PCR, *TRI* genes

## Abstract

*Fusarium* mycotoxins, deoxynivalenol (DON) and nivalenol (NIV) act as virulence factors and are essential for symptom development after initial infection in wheat. To date, 16 genes have been identified in the DON biosynthesis pathway. However, a comparative gene expression analysis in different chemotypes of *Fusarium graminearum* in response to *Fusarium* head blight infection remains to be explored. Therefore, in this study, nine genes that involved in trichothecene biosynthesis were analyzed among 3-acetyldeoxynivalenol (3-ADON), 15-acetyldeoxynivalenol (15-ADON) and nivalenol producing *F. graminearum* strains in a time course study. Quantitative reverse transcription polymerase chain reaction revealed that the expression of all examined *TRI* gene transcripts initiated at 2 days post-inoculation (dpi), peaked at three to four dpi and gradually decreased at seven dpi. The early induction of *TRI* genes indicates that presence of high levels of *TRI* gene transcripts at early stages is important to initiate the biosynthetic pathway of DON and NIV. Comparison of gene expression among the three chemotypes showed that relative expression of *TRI* genes was higher in 3-ADON producing strains compared with 15-ADON and NIV strains. Comparatively higher levels of gene expression may contribute to the higher levels of DON produced by 3-ADON strains in infected grains.

## Introduction

*Fusarium* head blight (FHB) is one of the major economically important fungal diseases in wheat, barley, corn, and other small grains worldwide. Wheat yield losses of up to 50% have been reported in North America due to FHB (McMullen et al., 1997; Goswami and Kistler, 2005). One of the major concerns of FHB is the contamination of infected grains with *Fusarium* mycotoxins. *Fusarium* mycotoxins represent the largest group of mycotoxins, which contains more than 140 known metabolites such as trichothecenes, zearalenone and fumonisins (Yazar and Omurtag, 2008; Sobrova et al., 2010). Among these mycotoxins, trichothecenes are one of the major *Fusarium* mycotoxins synthesized mainly by the members in the *Fusarium graminearum* species complex (FGSC), *F. culmorum*, *F. sprotrichioides* and *F. poae* (Desjardins et al., 1993; Foroud and Eudes, 2009; Wang et al., 2011). The fungi in the FGSC have the potential to devastate a crop by reducing grain quality and quantity. After *Fusarium* infection, the grains become contaminated with trichothecene mycotoxins such as deoxynivalenol (DON), produced by the pathogen, making the crop unsuitable for food and feed. Trichothecenes produced by *Fusarium* spp. act as virulence factors in wheat plants. It has been reported that DON is important for the spread of *F. graminearum* beyond the point of infection within the host plant. Proctor et al. (1995) have shown that *TRI5*^-^ mutants have reduced virulence compared to wild type strains in Wheat and Rye cultivars suggesting that trichothecene production contributes to the virulence of *F. graminearum*. Non-DON producing strains of *F. graminearum* can initiate the infection, but not spread within the host tissue (Proctor et al., 1995; Bai et al., 2002). A study done by Diamond et al. (2013) found that DON is capable of inhibiting the apoptosis–like programmed cell death in *Arabidopsis* cell cultures subjected to heat stress.

So far, 16 genes have been characterized in the DON biosynthesis pathway. These genes reside at four different loci on different chromosomes; the core *TRI* cluster consists of 12 genes located on chromosome 2, the *TRI1-TRI16* loci on chromosome 1, *TRI101* on chromosome 4, and *TRI15* on chromosome 3, respectively (Gale et al., 2005; Alexander et al., 2009; Merhej et al., 2011). The first step in the DON biosynthesis pathway consists of the cyclization of the initial substrate, farnesyl pyrophosphate (FPP) to produce non-toxic trichodiene, by the trichodiene synthase enzyme encoded by *TRI5* gene (Hohn and Beremand, 1989). The next nine reactions in the pathway are mediated by the enzymes encoded by *TRI4*, *TRI101*, *TRI11* and *TRI3*, respectively. These reactions lead to the formation of calonectrin, which serves as a substrate for the production of 3-ADON, 15-ADON and 4-acetylnivalenol (4-ANIV) (Alexander et al., 2009; Foroud and Eudes, 2009; Merhej et al., 2011). The genes *TRI7* and *TRI13* are functional only in *F. graminearum* strains that are capable of producing NIV (Brown et al., 2001; Lee et al., 2002). The enzymes encoded by *TRI7* and *TRI13* genes mediate two common steps following calonectrin. In nivalenol producing *F. graminearum* strains, the pathway continues with the product of *TRI1* to produce 4-ANIV and the final step mediated by *TRI8* to give NIV. The *TRI7* and *TRI13* genes are not active in DON producers; therefore, DON biosynthesis proceeds directly from calonectrin with the enzymes encoded by *TRI1* gene (McCormick and Alexander, 2002; Alexander et al., 2011; Merhej et al., 2011). The formation of 3-ADON or 15-ADON is strain specific and decided by the esterase coding sequence of *TRI8* gene (Alexander et al., 2011). To date, limited research has been done on expression of *TRI* genes in different chemotypes of *F. graminearum* during wheat colonization.

Among the different *TRI* genes, *TRI5* gene has received more attention and so far the majority of studies have focused on the expression of the *TRI5* gene during *Fusarium*-wheat colonization. A study done by Hallen-Adams et al. (2011) examined the expression of the *TRI5* gene during wheat spike infection of susceptible and resistant cultivars and susceptible cultivars treated with strobilurin fungicides. The highest expression of the *TRI5* gene was observed at the infection front. Gardiner et al. (2009) reported that *TRI5* gene is strongly expressed in the rachis tissue of wheat. In this study they used a *F. graminearum* strain constructed by fusing a green fluorescent protein (GFP) marker to the promoter of *TRI5* gene. Zhang et al. (2009) examined the expression of the *TRI5* gene between carbendazim-resistant and sensitive *F. graminearum* in shake culture and reported a significant exponential relationship between trichothecene production and *TRI5* gene expression. More recently Lee et al. (2014) compared the expression of *TRI* cluster genes in DON vs. NIV producing *F. graminearum* strains in liquid cultures. No study has been done to compare the level of expression of *TRI* genes in different chemotypes of *F. graminearum* during wheat colonization.

Therefore, in this study we have compared the level of expression of nine *TRI* genes in 3-ADON, 15-ADON and NIV-producing *F. graminearum* strains in a time course study both in resistant and susceptible wheat cultivars. The objective of this study was to evaluate the chemotype specific gene expression patterns in trichothecene biosynthesis related genes in different chemotypes of *F. graminearum* during wheat infection and colonization.

## Materials and Methods

### Greenhouse Experiment and RNA Isolation

Two wheat cultivars with different levels of resistance to *Fusarium* head blight (FHB) were used in this study. A spring wheat cultivar, Roblin, which is highly susceptible (S) to FHB, and a FHB moderately resistant (MR) cultivar, Carberry, with resistance originating from the Chinese cultivar Sumai3 were used in the study. The Chinese cultivar Sumai3 have both Type I and II FHB resistance (Bai and Shaner, 1994). To prepare inoculum, two *F. graminearum* strains from each chemotype were cultured on Spezieller Nährstoffarmer agar (SNA) medium (0.2 g glucose, 0.2 g sucrose, 1 g KH_2_PO_4_, 1 g KNO_3_, 0.25 g MgSO_4_.7H_2_O, 0.5 g KCl, 14 g technical agar in 1 L of distilled water). *F. graminearum* strains used in this study were consisted of; Q-06-11 (designated as: 3-ADON1, isolated from wheat in Canada), A6-06-01 (3-ADON2, isolated from wheat in Canada), PH1 (15-ADON1, isolated from wheat in the USA), M2-06-02 (15-ADON2, isolated from wheat in Canada), W52516 (NIV1, isolated from maize in China) and W56604 (NIV2, isolated from maize in China). To produce liquid inoculum, 1.5 L of carboxymethyl cellulose (CMC) liquid media (15 g CMC, 1 g NH_4_NO_3_, 1 g KH_2_PO_4_ monobasic, 5 g MgSO4.7H_2_O, 1 g yeast extract in 1 L of distilled water) was prepared and four SNA media (Leslie and Summerell, 2006) plates from each strain were divided into sections and added into each flask. Seven days after incubation at 25°C under fluorescent light, the number of conidia per milliliter was determined by using a haemocytometer. The final conidial concentration was adjusted to 50,000 conidia/mL using distilled water. Seeds of spring wheat cultivars; Carberry and Roblin were planted in 15-cm plastic pots and maintained at 22–24°C in the greenhouse at the Department of Plant Science, University of Manitoba, Winnipeg, MB, Canada. Inoculations were conducted at 30–50% anthesis. A 10 μL of *F. graminearum* suspension (50,000 conidia/mL) was injected between the palea and lemma of spikelets per each spike according to the protocol described by Cuthbert et al. (2006). Five biological replicates for each strain and time point were conducted following a complete randomized design. Four to five spikes were inoculated per plant. FHB disease severity (DS) ratings were taken at 2, 3, 4, 7, 10, and 14 days post-inoculation (dpi) using the FHB disease scale by Stack and McMullen (1995). FHB DS readings were taken from five inoculated spikes for each replicate. The inoculated spikes were sampled at 2, 3, 4, 7, 10, and 14 dpi and stored at -80°C freezer until RNA isolation. The mock inoculations were made using distilled water in both Roblin and Carberry for all time points. The inoculated spikes from five replicates were pooled and ground into fine powder in liquid nitrogen using a mortar and pestle. Total RNA was isolated using TRIzol^®^ reagent (Invitrogen Life Technologies, Carlsbad, CA, USA) according to manufacturer’s instructions. Extracted RNA was quantified using the NanoDrop 3300 (Thermo Scientific Inc., Wilmington, DE, USA). The integrity of RNA was analyzed using 1% agarose gel electrophoresis. To remove any DNA contaminations, RNA was treated with TURBO^TM^ DNaseI (Invitrogen Life Technologies, Carlsbad, CA, USA) before cDNA synthesis. The first strand of cDNA was synthesized from 2 μg total RNA as the template using SuperScript^TM^ III First-Strand Synthesis System for reverse transcription-polymerase chain reaction (Invitrogen Life Technologies, Carlsbad, CA, USA).

### FDK, DON and NIV Analysis

Kernels were harvested from inoculated spikes from both cultivars Carberry and Roblin, at 14 dpi. The percentage of *Fusarium* damaged kernels (FDK) was estimated by taking a pooled sample of 10 g from all replicates. The same kernels used for FDK analysis were used for DON or NIV analysis. Wheat kernels of each strain were pooled, ground and analyzed by Veratox^®^ DON 5/5 kit (product no: 8331, Neogen Corp., Lansing, MI, USA) for DON analysis. NIV analysis was done using GC-MS according to the protocol described by Tittlemier et al. (2013).

### Quantitative Reverse Transcription PCR

A total of nine genes (*TRI4*, *TRI5*, *TRI6*, *TRI3*, *TRI8*, *TRI101*, *TRI9*, *TRI12* and *FPP*) in the DON biosynthetic pathway were examined using quantitative reverse transcription PCR (qRT-PCR). The level of expression of each gene was analyzed using a set of gene specific primers as described by Lee et al. (2014). As a house-keeping gene, translation elongation factor 1 alpha (*EF-1α*) from *F. graminearum* was selected (Kim and Yun, 2011). qRT-PCR reactions were performed in a CFX96 Touch^TM^ Real Time PCR Detection System (Bio-Rad, Hercules, CA, USA) according to the protocol described by Lee et al. (2014). The qRT-PCR reaction cycles were consisted of initial denaturation at 95°C for 3 min, followed by 45 cycles at 95°C for 10 s, 60°C for 20 s, 72°C for 20 s, and finally 95°C for 10 s and 65°C for 5 s. The qRT-PCR reaction mixture contained 10 μL of 2× iQ SYBR^®^ Green Supermix consisted of SYBR^®^ Green I dye, 50 U/ml iTaq^TM^ DNA polymerase, 0.4 mM each dNTPs, 6 mM MgCl_2_, 40 mM Tris-HCl (pH 8.4), 100 mM KCl, 20 nM fluorescein and stabilizers (Bio-Rad, Hercules, CA, USA), 0.5 μL of each primer (10 pM), 1 μL of template cDNA (10 ng), and RNase free water to a final volume of 20 μL. Quantification values were analyzed using the Bio-Rad CFX Manager v1.6, and the threshold cycle (Ct) values were determined. In all reactions, a non-template control (NTC) was set up to avoid any DNA contaminations in the reaction mixtures. Each reaction sample was amplified three times and final Ct values were calculated as an average of three replicates. The relative transcript abundance of the target genes was determined by the Pfaffl method (Pfaffl et al., 2002). qRT-PCR primer amplification efficiency was determined using the Ct slope method. In this method, serial dilutions of the template were prepared and Ct values were determined. Then a standard curve was generated by plotting the Ct values against the log cDNA concentrations. The amplification efficiency (E) of each primer was determined using the formula E = 10^-1/slope^. The percent amplification efficiency was determined using the formula %E = (E-1) ^∗^100%. The percent amplification efficiency of all genes were ranged between 95.6 and 101.3%.

### Statistical Analysis

Analysis of variance (ANOVA) for FHB DS at seven and 14 dpi was performed using the PROC Mixed procedure of SAS software (SAS version 9.3, SAS Institute Inc., Cary, NC, USA). Cultivar, strain and cultivar*strain were considered as fixed effects. The Bonferroni method was used to compare statistically significant differences in least squares (LS) means of all variables. The type 3 test of fixed effects was determined and those with *p* ≤ 0.05 were considered significant.

## Results

**FIGURE 1:**
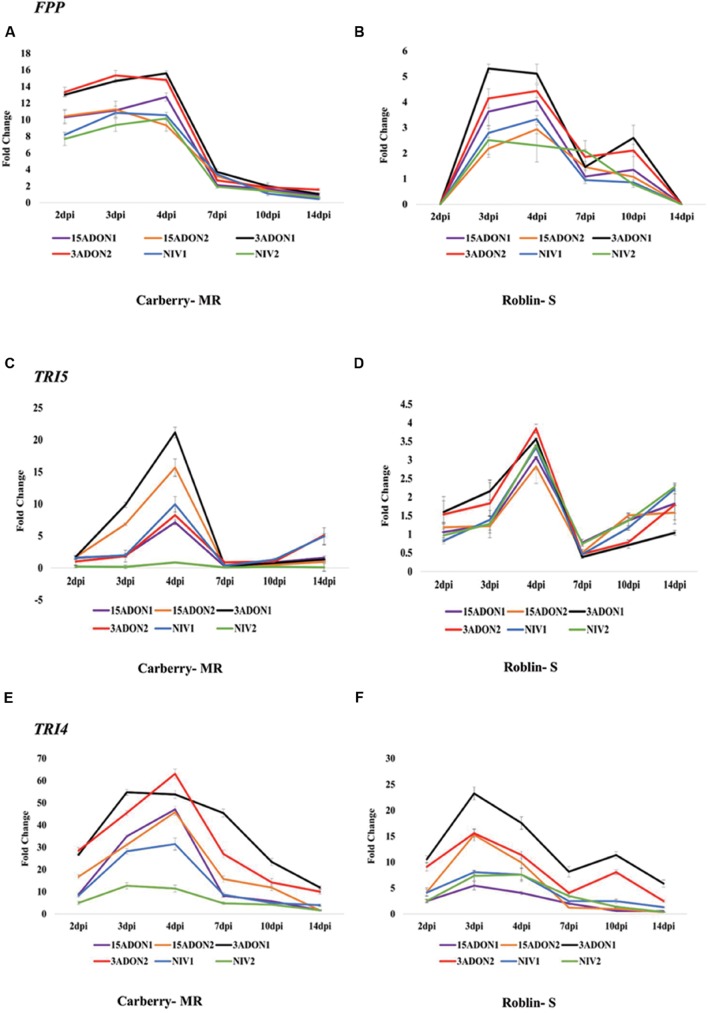
**Fold change in transcript levels of farnesyl pyrophosphate synthase (*FPP*) gene in cultivar Carberry **(A)** and cultivar Roblin **(B)**, trichodiene synthase (*TRI5*) gene in cultivar Carberry **(C)** and cultivar Roblin **(D)** and trichodiene oxygenase (*TRI4*) gene in cultivar Carberry **(E)** and cultivar Roblin **(F)**.** All data were normalized to the *EF-1α* expression level. Values are means ± SE of three replicates.

Nine genes from the *F. graminearum* trichothecene biosynthesis pathway, *TRI4*, *TRI5*, *TRI6*, *TRI3*, *TRI8*, *TRI101*, *TRI9*, *TRI12* and *FPP* along with the housekeeping gene *EF-1α*, were selected for gene expression analysis. Each selected gene was analyzed by quantitative reverse transcription PCR to examine the changes in transcript levels at different time intervals post-inoculation. Accumulation of *TRI* gene transcripts initiated as early as 2 dpi in most strains. Significant differences were observed for cultivar, strain, and dpi for all the analyzed genes. The qRT-PCR analysis showed that the *FPP* transcript accumulation initiated at 2 dpi, peaked at 3–4 dpi and rapidly decreased at 7 dpi in MR cultivar Carberry (**Figure 1A**). A similar transcript accumulation pattern was observed in the susceptible (S) cultivar Roblin, however, at 10 dpi there was a slight increase in transcript accumulation in 3-ADON1, 3-ADON2 and 15-ADON1 strains and then gradually decreased at 14 dpi (**Figure 1B**). In both MR cultivar and S cultivar, the abundance of *FPP* transcripts was higher in 3-ADON producing *F. graminearum* strains than 15-ADON and NIV producing strains at most time points.

**FIGURE 2:**
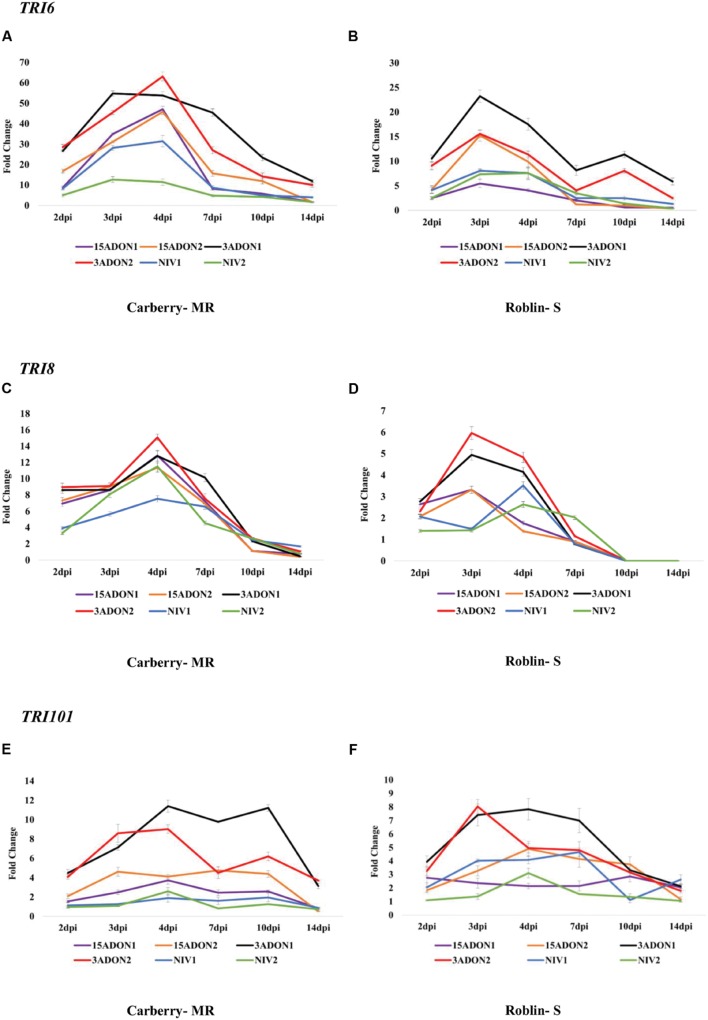
**Fold change in transcript levels of transcription factor *TRI6* gene in cultivar Carberry **(A)** and cultivar Roblin **(B)**, trichothecene 3-*O*-esterase (*TRI8*) gene in cultivar Carberry **(C)** and cultivar Roblin **(D)** and trichothecene 3-*O*-acetyltransferase (*TRI101*) gene in cultivar Carberry **(E)** and cultivar Roblin **(F)****.

The qRT-PCR analysis for *TRI5* showed that, transcript accumulation initiated at 2 dpi and peaked at 4 dpi and rapidly decreased by day 7. In contrast to the expression pattern of *FPP* gene, *TRI5* gene expression increased again after 7 dpi in both cultivars (**Figures 1C,D**). Similar to *FPP* and *TRI5* genes, accumulation of *TRI4* transcripts initiated at 2 dpi, peaked at 3–4 dpi and started decreasing after day 4 in both cultivars (**Figures 1E,F**). However, in cultivar Roblin (S), transcript accumulation again peaked at 10 dpi in 3-ADON producing strains. The accumulation of *TRI6* transcripts also initiated at 2 dpi, peaked at 4 dpi and gradually decreased after day 4 in cultivar Carberry (MR) (**Figure 2A**). In cultivar Roblin (S), transcript accumulation initiated at 2 dpi, peaked at 3 dpi and start decreasing after day 3 (**Figure 2B**). In 3-ADON producing strains the level of gene expression again peaked at 10 dpi. For *TRI8* gene, transcript accumulation was initiated at 2 dpi and peaked at 4 dpi in all strains in cultivar Carberry (MR) (**Figure 2C**). In cultivar Roblin (S), transcript accumulation peaked at 3 dpi in 3-ADON and 15-ADON strains whereas for NIV strains it was at 4 dpi (**Figure 2D**).

**FIGURE 3:**
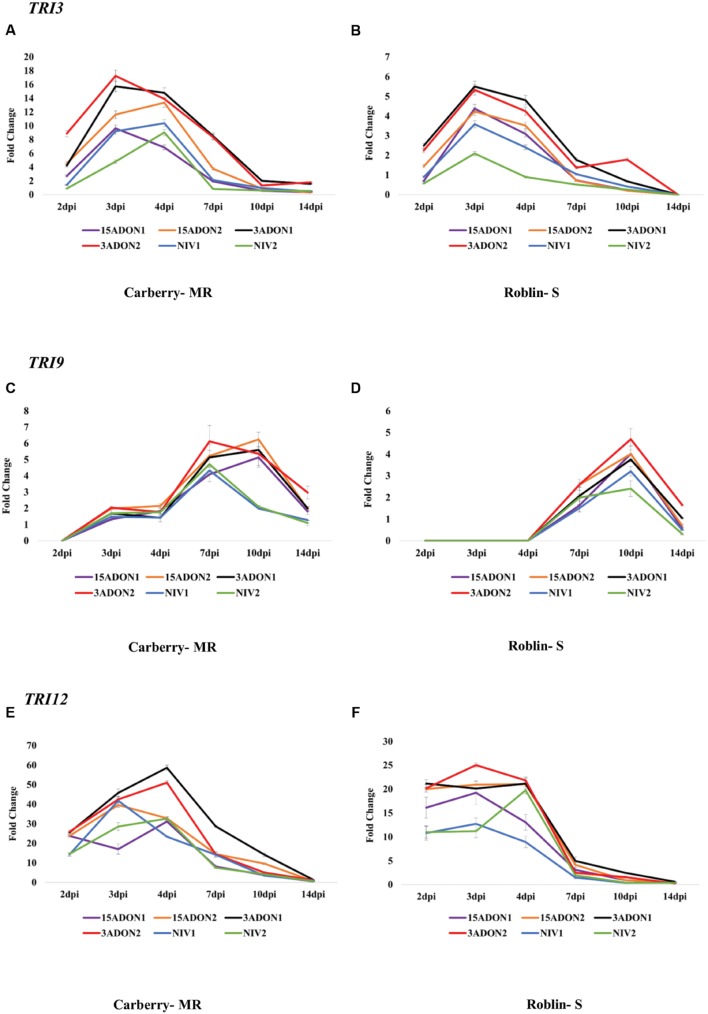
**Fold change in transcript levels of trichothecene 15-*O*-acetyltransferase (*TRI3*) gene in cultivar Carberry **(A)** and cultivar Roblin **(B)**, *TRI9* gene in cultivar Carberry **(C)** and cultivar Roblin **(D)** and *TRI12* gene in cultivar Carberry **(E)** and cultivar Roblin **(F)****.

The expression of the *TRI101* gene initiated at 2 dpi and remained relatively constant for 15-ADON1, 15-ADON2, NIV1 and NIV2 strains during the early time intervals at 2–7 dpi (no distinct peaks were observed) and started decreasing at 10 dpi in MR cultivar, Carberry (**Figure 2E**). In 3-ADON1, peaks were observed at 4 and 10 dpi. A similar pattern was observed in S cultivar Roblin, however, a distinct peak was observed for 3-ADON2 at 3 dpi (**Figure 2F**). Similar to other genes, the accumulation of *TRI3* transcripts initiated at 2 dpi and peaked at 3–4 dpi in most strains in both cultivars; however, the transcript abundance started decreasing at 4 dpi (**Figures 3A,B**). Despite the earlier induction (2 dpi), the level of expression of *TRI*9 gene peaked at 7–10 dpi (in cultivar Carberry) and 10 dpi (in cultivar Roblin) and gradually decreased in both cultivars starting at 10 dpi (**Figures 3C,D**). Transcript accumulation of the *TRI12* gene also initiated at 2 dpi, peaked at 4 dpi and gradually decreased at 7 dpi in most of the strains (**Figures 3E,F**). Based on the qRT-PCR data, *F. graminearum* 3-ADON strains showed a higher level of *TRI* gene expression compared to the other strains for genes *FPP*, *TRI3*, *TRI4*, *TRI6*, *TRI8*, *TRI12*, and *TRI101*, at most time points except for *TRI5* gene in MR cultivar and *TRI9* gene. In *TRI5* gene, 3-ADON1 and 15-ADON2 strains showed higher levels of expression than other *F. graminearum* strains in MR cultivar Carberry. The level of gene expression in 15-ADON and NIV producing strains showed no specific pattern of higher or lower expression. In some genes and time points the level of transcript accumulation was higher in 15-ADON strains and lower in NIV strains and vice versa. Among the analyzed genes, the highest abundance of transcripts was observed for *TRI4* and *TRI12* genes for all the examined strains (**Figures 1E,F** and **3E,F**). Our data showed that relative expression of *TRI* genes was significantly higher in wheat cultivar Carberry (MR) compared with Roblin (S).

**FIGURE 4:**
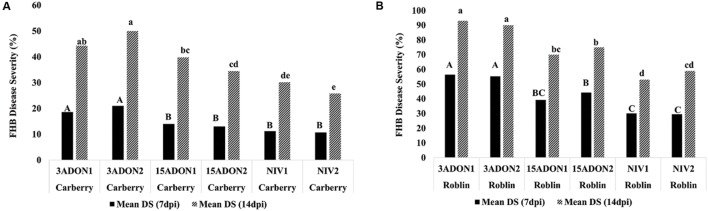
**Mean Fusarium head blight disease severity in **(A)** moderately resistant cultivar Carberry and **(B)** susceptible cultivar Roblin after inoculating with different chemotypes of *Fusarium graminearum* at 7 and 14 days post-inoculation.** Means with the same letters for *Fusarium* head blight disease severity are not significantly different.

**FIGURE 5:**
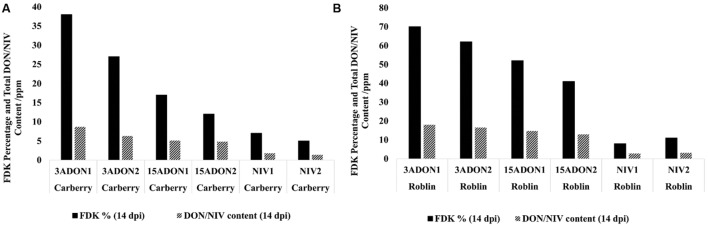
**Fusarium damaged kernel (FDK) percentage and total terminal deoxynivalenol (DON) or nivalenol (NIV) content in **(A)** moderately resistant cultivar Carberry and **(B)** susceptible cultivar Roblin after inoculating with different chemotypes of *Fusarium graminearum* at 14 days post-inoculation**.

The FHB DS was analyzed at 7 and 14 dpi, terminal FDK and DON/NIV content were analyzed at 14 dpi. When FHB DS was considered, there were significant differences between the cultivars and among the strains. The two-way interaction cultivar^∗^strain was significantly different (**Table 1**). The highest FHB DS was shown by cultivar Roblin inoculated by 3-ADON strains followed by 15-ADON and NIV strains. FHB DS caused by 3-ADON strains was significantly different from the 15-ADON producing strains and NIV strains. A similar trend was observed in the MR cultivar Carberry, however, the FHB symptom development was slower than in cultivar Roblin which is highly susceptible to FHB (**Figures 4A,B**). The percentage of FDK was higher in cultivar Roblin than in cultivar Carberry (**Figures 5A,B**). Similarly, a higher total DON content was observed in cultivar Roblin inoculated with 3-ADON strains than the 15-ADON strains (**Figures 5A,B**). Cultivars inoculated with 3-ADON strains showed higher levels of FDK and DON content than 15-ADON strains. NIV producing strains showed the lowest FDK percentage and toxin contamination.

**Table 1:** Analysis of variance (ANOVA) table for cultivar, strain and their interaction for *Fusarium* head blight disease severity at 7 and 14 days post-inoculation.

Days post-inoculation	Source	DF	MS	F value	Pr > F
7 dpi	Cultivar	1	34556	694.69	<0.0001
	Strain	5	1858.88	37.37	<0.0001
	Cultivar^∗^Strain	5	478.35	9.62	0.0001
	Rep	14	60.10	1.20	0.2806
	Error	154	49.74		
14 dpi	Cultivar	1	59405	585.26	<0.0001
	Strain	5	4323.23	42.59	<0.0001
	Cultivar^∗^Strain	5	2804.13	5.53	0.0001
	Rep	14	58.06	0.57	0.8836
	Error	154	101.5		


## Discussion

The objective of this study was to identify the potential chemotype-specific gene expression patterns of the *TRI* genes during wheat- *F. graminearum* infection and colonization. The expression of most *TRI* genes required for trichothecene production in *F. graminearum* were strongly induced at early time points after infection (i.e., 2–4 dpi) and the expression levels gradually decreased at 7 dpi. Also 3-ADON producing strains showed a comparatively higher level of gene expression than 15-ADON and NIV producing strains, confirming their ability to produce higher amounts of toxin in infected wheat kernels.

Deoxynivalenol biosynthesis related gene expression profiling indicated that, the expression of most *TRI* genes were initiated at 2 dpi. This shows that a high level of *TRI* transcript accumulation is essential for initiating the biosynthetic pathway of DON or NIV during wheat infection and colonization. The early expression patterns of five *TRI* genes along with *FPP* gene (*TRI4*, *TRI*5, *TRI*6, *TRI8* and *TRI3*) strongly suggested that *TRI6* gene which encodes a transcriptional regulator, positively regulates the expression of other *TRI* genes in the DON biosynthesis pathway. Similar observations have reported by Lee et al. (2014) in liquid culture media. The level of *TRI* gene expression was significantly different among the three chemotypes analyzed. The level of expression of most of the examined genes was higher in 3-ADON producing strains in both cultivars compared to the 15-ADON producing strains and NIV producing strains. It has been reported that 3-ADON strains produce more trichothecenes than 15-ADON and NIV strains (Ward et al., 2008). Therefore, the higher levels of expression of trichothecene biosynthesis related genes in 3-ADON producing strains during colonization may mediate the production of high amounts of toxins. According to the total DON content at 14 dpi, kernels infected with 3-ADON strains showed higher total DON content than the 15-ADON strains in both cultivars. Also, in this study the level of transcript accumulation of *TRI4* gene was comparatively higher than other genes (except for *TRI12* gene). The *TRI4* gene regulates multiple steps (four steps) in the trichothecene biosynthesis pathway (McCormick et al., 2006). The accumulation of *TRI4* transcripts in higher amounts could be explained by the involvement of this gene in multiple steps during trichothecene production. The level of expression of the *TRI12* gene initiated at 2 dpi, peaked at 4 dpi and gradually decreased at 7 dpi, which was similar to the other analyzed *TRI* genes in the present study. It has been reported that *TRI12* gene encodes for trichothecenes efflux pump, which gives self-protection for the fungus from the produced trichothecenes (Alexander et al., 1999). Therefore, coherent gene expression patterns of *TR112* genes with other analyzed *TRI* genes further supports the role of *TRI12* gene as a self-protector against the produced trichothecenes.

The level of expression of *TRI* genes was significantly higher in the MR cultivar Carberry than in the S cultivar Roblin. Similar results have been reported in other studies (Boddu et al., 2007; Brown et al., 2011; Hallen-Adams et al., 2011). Still there is no clear reason to explain the higher levels of expression in trichothecene biosynthesis genes in MR cultivar compared to the S cultivar. However, when we analyzed the total DON content at 14 dpi it was higher in the susceptible cultivar Roblin than in the MR cultivar Carberry. DON is a virulence factor in wheat, necessary for the spread of the fungus beyond the point of infection (Proctor et al., 1995; Bai et al., 2002; Audenaert et al., 2013). The above contrary observations between the trichothecene biosynthesis gene expression and terminal DON content in MR and S cultivars can be explained as follows: *F. graminearum* enters the plant either through inoculation or natural infection; in the first stage, it grows biotrophically at the point of infection and start producing DON (Audenaert et al., 2013). Then the fungus becomes more aggressive and attempts to grow into adjacent spikelets. However, the resistance mechanisms in R or MR cultivars prevent the fungus invasion from the point of infection. In order to overcome the resistance and spread further from the point of infection, fungus increases DON production as DON acts as a virulence factor in wheat. Finally, to increase the DON production, the fungus increases the level of expression of DON biosynthesis related genes in R or MR cultivars compared to S cultivars. Investigations are in progress to further understand the reasons for higher levels of *TRI* gene expression in MR cultivars than in S cultivars.

Although the level of expression of DON biosynthetic genes were higher in the MR cultivar than in the S cultivar the final DON content is higher in the S cultivar. It has been reported that during *Fusarium* infection there is a broad expression of genes related to the DON detoxification process (Muhovski et al., 2012). This may explain the low levels of DON contamination in MR cultivar compared to S cultivar. Gene expression studies have shown that the expression of DON detoxification transcripts such as UDP-glycosyltransferase family (UGTs), CYP450s, ABC transporters and multidrug resistance-associated protein (MRP) were more highly abundant in FHB resistant cultivars than in susceptible cultivars during *Fusarium* infection (Muhovski et al., 2012; Al-Taweel et al., 2014; Kosaka et al., 2015). Therefore, it can be hypothesized that, although the level of *TRI* gene expression is higher in the MR cultivar, the resistance mechanisms within the cultivar can more efficiently detoxify the produced DON than the susceptible cultivar.

This study provides evidence on the chemotype specific gene expression patterns in the DON biosynthesis pathway during wheat infection and colonization. The results from this study indicated that 3-ADON producing strains showed higher levels of gene expression compared to 15-ADON and NIV producing strains. However, use of only two strains representing a chemotype may not be sufficient to draw definitive conclusions. Therefore, this study suggests the use of more strains from each chemotype group to gain a more comprehensive understanding of chemotype specific gene expression patterns during *F. graminearum* infection and colonization.

## Author Contributions

CA performed the research, coordinated the experimental part of the project and wrote the manuscript. WF supervised the research project and critically reviewed the manuscript.

## Conflict of Interest Statement

The authors declare that the research was conducted in the absence of any commercial or financial relationships that could be construed as a potential conflict of interest.
